# Artificial Intelligence and Behavioral Science Through the Looking Glass: Challenges for Real-World Application

**DOI:** 10.1093/abm/kaaa095

**Published:** 2021-01-08

**Authors:** Pol Mac Aonghusa, Susan Michie

**Affiliations:** 1 Health and Social Care Research Group, IBM Research, Dublin, Ireland; 2 Centre for Behaviour Change, University College London, London, UK

**Keywords:** Artificial intelligence, Machine learning, Behavior change, Evidence synthesis, Prediction algorithms, Interventions

## Abstract

**Background:**

Artificial Intelligence (AI) is transforming the process of scientific research. AI, coupled with availability of large datasets and increasing computational power, is accelerating progress in areas such as genetics, climate change and astronomy [NeurIPS 2019 Workshop Tackling Climate Change with Machine Learning, Vancouver, Canada; Hausen R, Robertson BE. Morpheus: A deep learning framework for the pixel-level analysis of astronomical image data. Astrophys J Suppl Ser. 2020;248:20; Dias R, Torkamani A. AI in clinical and genomic diagnostics. *Genome Med*. 2019;11:70.]. The application of AI in behavioral science is still in its infancy and realizing the promise of AI requires adapting current practices.

**Purposes:**

By using AI to synthesize and interpret behavior change intervention evaluation report findings at a scale beyond human capability, the HBCP seeks to improve the efficiency and effectiveness of research activities. We explore challenges facing AI adoption in behavioral science through the lens of lessons learned during the Human Behaviour-Change Project (HBCP).

**Methods:**

The project used an iterative cycle of development and testing of AI algorithms. Using a corpus of published research reports of randomized controlled trials of behavioral interventions, behavioral science experts annotated occurrences of interventions and outcomes. AI algorithms were trained to recognize natural language patterns associated with interventions and outcomes from the expert human annotations. Once trained, the AI algorithms were used to predict outcomes for interventions that were checked by behavioral scientists.

**Results:**

Intervention reports contain many items of information needing to be extracted and these are expressed in hugely variable and idiosyncratic language used in research reports to convey information makes developing algorithms to extract all the information with near perfect accuracy impractical. However, statistical matching algorithms combined with advanced machine learning approaches created reasonably accurate outcome predictions from incomplete data.

**Conclusions:**

AI holds promise for achieving the goal of predicting outcomes of behavior change interventions, based on information that is automatically extracted from intervention evaluation reports. This information can be used to train knowledge systems using machine learning and reasoning algorithms.

## Teaser

Artificial intelligence holds promise for achieving the goal of predicting outcomes of behavior change interventions, using information automatically extracted from intervention evaluation reports.

## Introduction

Artificial intelligence (AI) is currently in a boom. Technological advances in AI have advanced natural language and vision processing to the point where driverless cars are a reality and speaking personalized assistants have become commonplace. Adoption of AI in human-centric practices is still at a relatively early stage. We explore core challenges facing AI adoption in a human-centric discipline, behavioral science, through the practical lens of lessons learned during a 4 year research collaboration between behavioral scientists and computer scientists, the Human Behaviour-Change Project (HBCP).

The HBCP is a collaboration of behavioral scientists and technologists using cutting-edge AI to synthesize and interpret evidence automatically extracted from behavioral intervention research studies as a basis for predicting intervention outcomes [1, 2]. Beginning with randomized trials of behavior change interventions for smoking cessation, the HBCP system is intended to eventually synthesize knowledge extracted from many sources. The system is artificially intelligent in the sense that it may also propose new hypotheses about behavioral interventions and their expected effectiveness. In effect, the system enables the prediction of outcomes of different intervention scenarios in different populations, settings, and target behaviors.

Predicting outcomes is of great interest to policy makers, planners, and practitioners who seek answers to complex questions along the lines of: “What works, compared with what, for what behaviors, how well, for how long, with whom, in what setting, and why?” [1]. This is a complex question and not all users of the Knowledge System produced by the HBCP will want to know about all aspects of this question. There may be constraints to users’ questions, for example, in terms of target population or behavior; on the other hand, they may be very open to a range of modes of delivery of the intervention and setting in which the intervention is delivered. To address this question, the vast and accelerating rate of evidence generated in the behavior change literature needs to be synthesized at scale and at speed, beyond the capability of even large teams of highly trained evidence reviewers. This is where AI comes in.

How can AI make sense of this information presented in very variable language and formats, for example, data tables that are laid out differently according to journal. There is frequently not enough information available in research reports to analyze the complex interactions between effects and various aspects of interventions, populations, and settings.

A framework of standard structures and terminology is needed to draw together disparate evidence in a way that is “AI-friendly.” In the HBCP, having a shared reference framework denoting how knowledge about interventions, outcomes, and the relationships between them should be formally represented as “ontologies” has been critical to the project [3]. Ontologies help formalize common understanding across disciplines and between academics and technologists. Ontologies are also commonly used in AI and so provide a natural building block for AI systems. The upper level of the Behavior Change Intervention Ontology (BCIO) can be seen alongside a methods paper explaining how this was developed [4, 5].

## The Human Behaviour-Change Project

Development of the HBCP system follows what is commonly called a “supervised learning” approach in the AI literature. In a supervised learning approach, a “training set” of annotated types of information to be extracted is first prepared. Annotations can contain rich information about the examples, but it is useful to think of a minimal annotation as a standard text label describing what the annotated data are.

**Fig. 1.**
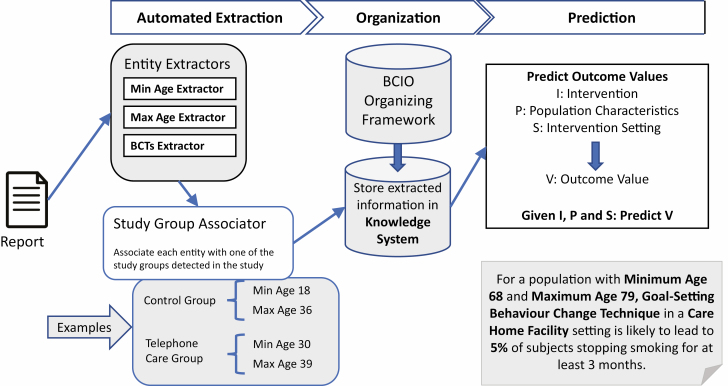
Conceptual illustration of the processing steps in the Human Behavior-Change Project.

Taking the expertly annotated training set as initial examples, the goal of the automated extraction phase of activity is to construct AI algorithms and tune them to automatically recognize and extract instances of the same types of annotated entities when presented with new reports of randomized controlled trials of behavioral interventions. The automated extraction phase is shown in Fig. 1, illustrating how entities are extracted and then matched to a study group detected in the report. When processing text appearing in behavioral reports created by humans, the process of extraction of information by AI algorithms is typically called Natural Language Processing (NLP). The term “machine learning” is commonly used in AI literature and, in this paper, to describe the process of constructing computer algorithms to perform tasks by learning patterns from examples.

The annotation and extraction activity phases were focused on two main challenges. The first was *detecting the presence* of entities for behavior change techniques referenced in the BCIO, such as *goal setting, problem solving*, or *action planning* in published study reports. The second was *extracting values* of attributes, such as *mean ages* and *genders* of participants. For detecting both the presence of entities and their values, the first step was for researchers to code each manuscript for the type of entity (e.g., age) and its attributes (e.g., mean age). These manuscripts were then used for AI training to automatically detect and extract the relevant information. The extracted information was organized according to the BCIO; for a detailed description, see [6]. Organizing extracted information is illustrated in Fig. 1, with the BCIO providing a common framework for structuring the extracted information in a consistent way.

Entities to be extracted from smoking cessation intervention reports were added to the system in three waves of 31, 26, and 24 entities, with outcomes of multiarm trials and intervention characteristics on a per-arm basis included in the second wave. The first wave of value extraction entities identified by the project team comprised: minimum age, maximum age, mean age, gender, effect size estimate, outcome value of the intervention, and outcome value of the comparison condition.

Extracted information is stored into an organized format suitable for computer processing referred to as the “Knowledge System” and illustrated in Fig. 1. The BCIO was used as a common structure for the knowledge acquired from intervention reports [4]. An ontology is a standardized representational framework providing a set of terms for the consistent description (or “annotation” or “tagging”) of data and information across disciplinary and research community boundaries [7]. A key benefit of having an ontology as a common framework is that extracted data from reports can be mapped to a consistent set of concepts provided by the ontology, regardless of how the data were originally expressed in reports. As a simple example, a range of ages for a population sample might be expressed as “teenager” in one report and stated as “between 13 and 19 years” in another. When mapping both expressions to the BCIO ontology, the individual expressions are automatically converted to a single consistent representation, such as (Min_Age = 11, Max_Age = 19) in the knowledge structure. In this way, computer programs accessing the knowledge structure will always see a single, consistent representation of age ranges regardless of original expression.

For the final prediction phase of activity, shown on the right of Fig. 1, large amounts of reliably extracted information from multiple reports are needed to extrapolate from study findings to predict outcomes of “scenarios.” Scenarios are combinations of entities that allow a user to understand intervention effects, such as aspects of the intervention content and delivery, its mechanisms of action, the target behavior, and its context, for example, population and setting. To implement scenarios, machine learning algorithms take subsets of extracted entities (e.g., those describing the population, settings, and behavior change techniques) and predict another entity (e.g., an outcome value) in response to user queries.

Users construct scenarios by describing intervention scenarios and having the system predict outcomes from the information in the Knowledge System. For example, a scenario might be “Predict the likely success of **Goal-Setting Behavior Change Intervention** for a population with **Minimum Age 68** and **Maximum Age 79** in a **Care Home Facility** setting.” In response to this query, as illustrated in Fig. 1, the system might reply that “For a population with **Minimum Age 68** and **Maximum Age 79**, a **Goal-Setting Behavior Change Intervention** is likely to lead to **5%** of subjects to stop smoking.” The system will also allow the user to explore the evidence the system relied on to make the prediction by displaying the original sources of data, for example, as an explanation for the prediction process. Providing insight into the prediction is an important step in building user trust in the system.

The first behavior change domain selected was smoking cessation; the second will be physical activity. Smoking cessation was selected as the first use case as it was judged to have more robust and homogeneous outcome measures, large numbers of high-quality trials and better standards for conducting and reporting trials than other behavioral domains. For further information, see the study website https://www.humanbehaviourchange.org/, the protocol [1], and the papers published in Wellcome Open Research https://wellcomeopenresearch.org/collections/humanbehaviourchange [2].

## The Evolving Role of AI in the HBCP

In the early stages of the project, much of the effort was invested in constructing NLP algorithms to automatically detect intervention entities and their attributes. State-of-the-art algorithms that perform well in the computer science research literature are often tuned to perform well for curated data sources used by the computer science community intended specifically to compare algorithm performance. As a result, algorithms can fail to generalize easily to addressing real-world questions. This is especially true where research reports use diverse expression as is the case for reports of behavior change interventions and their evaluations.

Initial assessment of automated extraction results reflected the variability of expression present in individual study reports. For example, the initial sensitivity (also known as true positive rate or recall) for extraction of gender values was >75%, whereas minimum, maximum, and mean age were 35–40% and outcome value was <10%. Automatically detecting the presence of behavior change techniques had similarly variable results. Action planning and providing information about health consequences, for example, achieved initial sensitivity of >90%, while feedback on behavior and reducing negative emotions had an initial sensitivity of 35–40%.

In a cross-disciplinary project, such as the HBCP, drawing on the knowledge of all parties is vital in progressing effectively. For example, it is relatively straightforward to teach an AI algorithm to find terms as they occur in documents to construct a document search engine. However, the objective of the HBCP is to predict the effectiveness of real-world interventions, which is a more complex task requiring the expertise of both behavioral and computer scientists. For tasks such as prediction, an algorithm must be trained to interpret the meaning behind terms like “youth,” teenager’, “adolescent,” and “young adult.” To transform each instance into readily computable elements, such as numeric ranges of ages, is largely a technical task requiring computer science expertise. The practical decision by the HBCP team was that annotating *numerical data* corresponding to terms such as age groups, rather than annotating *unstructured text* associated with these terms, was a more efficient strategy. Numeric terms have less variability compared to unstructured text, which could be defined and interpreted very differently depending on context. Using the former reduced the complexity of both annotation and subsequent automated extraction.

During the later stages of the third wave of annotation, enough information had been extracted from multiple reports to begin trialing intervention outcome prediction from automated knowledge synthesis. From its inception, the goal of the project was to automate the extraction of information from vast numbers of behavior change reports, making it faster and more accurate and, therefore, more cost effective. In addition, it did not set out to mimic expert human information extraction of each entity from every report but to use AI to circumvent this requirement. However, as the number of entities from the BCIO needing to be processed grew, maintaining a high level of accuracy of extracted values proved increasingly challenging.

The intention in the HBCP was that the large scale of data extracted would allow statistical imputation of missing or imprecise features in reports from statistical analysis of values gathered from many reports. By building a model of intervention scenarios from the Knowledge System, an AI prediction algorithm could use the model to predict outcomes for new intervention scenarios. In addition to answering users’ queries about behavior change interventions, the Knowledge System will provide as full an explanation as possible of how its prediction was arrived at, including the information drawn on and the reasoning processes.

While so far limited to the smoking cessation domain, the experience of the HBCP suggests that AI can help to generate new knowledge about the effectiveness of behavioral interventions. Currently being applied to physical activity, the technologies developed by the HBCP are intended to generalize to other behavioral domains. The ability to generate new knowledge while explaining the workings of the algorithms aligns with a fundamental design goal of modern AI, that is, to enable humans to readily interpret the results of AI calculations using familiar concepts [8].

## Implications for Behavioral Science

AI is already in use in behavioral science. Increasing numbers of sophisticated information extraction techniques are being used to analyze text at speed and organize extracted data. For example, they are being used to search for online literature and AI NLP tools are assisting with literature reviewing [9,10]. The goal of the HBCP is to go further in investigating how inference can be used to synthesize new knowledge for tasks such as prediction of intervention outcomes and generation of hypotheses about, for example, the mechanisms by which interventions have their effects. While this is still at an early stage in the HBCP, the implications for behavioral science are significant. For example, it should enable researchers to go much further than they can currently in identifying gaps in the literature for investigation by using the AI to identify scenarios where there is weak, conflicting, or missing evidence of the effectiveness of behavioral interventions. A policy maker or a behavior change professional could use the same AI to look for the interventions that may be the most effective for a scenario that has not actually been studied yet.

As we have learned in the HBCP, exploiting advanced capabilities of AI requires human effort to prepare the literature for processing. Computers prefer structure, while behavior change intervention evaluation reports tend to be written in very varied formats using heterogeneous terminology. Organizing key elements of reports into standardized forms enables them to be more readily readable by computers and seems an inevitable implication of widespread AI adoption. A word of reassurance though, this should not be interpreted as an attempt to restrict creativity by limiting expressivity. Consistent and clearly interpretable use of language should improve the human writer’s and reader’s ability to communicate novel ideas. In the HBCP, the BCIO provided a standard schema by using the consistent, scientific language of ontologies.

The BCIO was developed to characterize interventions, their contexts and evaluations. It currently includes more than 2,000 entities. Development has followed good practice [5, 11] involving the annotation of more than 500 reports and feedback from a group of ontology experts and many international behavioral science experts (see [4]).

The project has highlighted the need for simplified and standardized reporting; a spin-off has been the development of an ontology-based Paper Authoring Tool to enable authors to easily and efficiently report their randomized controlled trials clearly, comprehensively, and in computer readable form. The tool has been developed for the journal *Addiction* and is underpinned by a second spin-off, the nascent Addiction Ontology [12]. The current version of the Paper Authoring Tool provides a much greater degree of structure and comprehensiveness of reporting than is typically provided in research reports, as well as supporting users in adopting terms with defined meanings. This allows the system to prompt intelligently for information it “knows” is going to be needed in another part of the paper [12].

A further spin-off has been to apply ontological modeling to synthesize and integrate the plethora of overlapping and partial theories of behavior change, working with a large data set of theories identified in a multidisciplinary literature review [9, 13]. A methodology was developed and evaluated in a set of five frequently used theories [14] and then applied, in collaboration with the theory authors, to represent 76 behavioral theories in a precise and computable format [15].

The change of perspective from AI attempting to match exact human performance and, instead, exploiting the statistical inference capabilities of AI to augment human performance in the HBCP has broader implications for the behavioral science community. Predictions obtained from an AI will necessarily have to be explainable so that the researcher can interpret the results correctly for further research. Practitioners and policy makers need to understand the consequences of accepting or rejecting a prediction that might impact individual well-being. Building confidence in predictions based on statistical inference by complex and opaque AI algorithms means engaging the behavioral science community in discussion to decide how important AI governance issues, such as fairness, bias, accountability, and transparency, will be managed.

The BCIO consists of both an ontology of the behavior change intervention scenario and an ontology of its evaluation, including study design and risk of bias. A structure to organize this knowledge is key to providing an estimate of confidence in the predictions of the Knowledge System. Such an estimate of confidence will be an important part of engendering appropriate trust by users. The next phase of the HBCP will include building the user interface, conducting a parallel study into trust in AI, including appropriate trust in the HBCP Knowledge System, and an evaluation of the Knowledge System as a whole. The latter will include efficiency of computational process, user experience, and usefulness of predictions to address real-world problems.

In conclusion, the aim of this innovative and collaborative work is to bring AI together with behavioral science expertise to improve significantly our use of available data to make predictions about outcomes of behavior change interventions *to* inform policy and practice. We envision that the work will also draw attention to additional scientific work that could advance both computer and behavioral science and promote research collaborations across disciplines, as well as provide practical solutions to policy makers, planners, and practitioners.

## References

[1] Michie S, Thomas J, Johnston M, et al. The Human Behaviour-Change Project: Harnessing the power of artificial intelligence and machine learning for evidence synthesis and interpretation. Implement Sci. 2017;12:121. 29047393 10.1186/s13012-017-0641-5 PMC5648456

[2] Michie S, Thomas J, Mac Aonghusa P, et al. The Human Behaviour-Change Project: An artificial intelligence system to answer questions about changing behaviour [version 1; peer review: not peer reviewed]. Wellcome Open Res. 2020;5:122. 32566761 10.12688/wellcomeopenres.15900.1 PMC7287511

[3] Hastings J. Primer on ontologies. In Dessimoz C and Škunca N, eds. The Gene Ontology Handbook. New York, NY: Springer New York; 2017:3–13.

[4] Michie S, West R, Finnerty A, et al. Representation of behaviour change interventions and their evaluation: Development of the upper level of the behaviour change intervention ontology [version 1; peer review: 1 approved, 1 approved with reservations]. Wellcome Open Res. 2020;5:123. 33614976 10.12688/wellcomeopenres.15902.1 PMC7868854

[5] Wright A, Norris E, Finnerty A, et al. Ontologies relevant to behaviour change interventions: A method for their development [version 2; peer review: 1 not approved]. Wellcome Open Res. 2020;5:126. 33447665 10.12688/wellcomeopenres.15908.1 PMC7786424

[6] Bonin F, Finnerty A, Moore C, et al. HBCP corpus: A new resource for the analysis of behaviour change intervention reports. Paper presented at the 12th Language Resources and Evaluation Conference; Marseille, France; May 2020.

[7] Arp R, Smith B, Spear AD. Building Ontologies with Basic Formal Ontology. Cambridge: MIT Press; 2015.

[8] Lehman E, DeYoung J, Barzilay R, Wallace BC. Inferring which medical treatments work from reports of clinical trials. Paper presented at the 2019 Conference of the North American Chapter of the Association for Computational Linguistics: Human Language Technologies. Volume 1 (Long and Short Papers). Minneapolis, MN; June 2019.

[9] Michie S, West R, Campbell R, Brown J, Gainforth H. ABC of Behaviour Change Theories. London: Silverback Publishing; 2014.

[10] Marshall IJ, Johnson BT, Wang Z, Rajasekaran S, Wallace BC. Semi-automated evidence synthesis in health psychology: Current methods and future prospects. Health Psychol Rev. 2020;14:145–158. 31941434 10.1080/17437199.2020.1716198 PMC7029797

[11] Norris E, Marques M, Finnerty A, et al. Development of an Intervention Setting Ontology for behaviour change: Specifying where interventions take place [version 1; peer review: 2 approved]. Wellcome Open Res. 2020;5:124. 32964137 10.12688/wellcomeopenres.15904.1 PMC7489274

[12] West R. An online Paper Authoring Tool (PAT) to improve reporting of, and synthesis of evidence from, trials in behavioral sciences. Health Psychol. 2020;39:846–850. 32833486 10.1037/hea0000927

[13] Davis R, Campbell R, Hildon Z, Hobbs L, Michie S. Theories of behaviour and behaviour change across the social and behavioural sciences: A scoping review. Health Psychol Rev. 2015;9:323–344. 25104107 10.1080/17437199.2014.941722 PMC4566873

[14] West R, Michie S, Rubin GJ, Amlôt R. Applying principles of behaviour change to reduce SARS-CoV-2 transmission. Nat Hum Behav. 2020;4:451–459. 32377018 10.1038/s41562-020-0887-9

[15] Hale J, Hastings J, West R, et al. An ontology-based modelling system (OBMS) for representing behaviour change theories. Wellcome Open Res. 2020;5:177. 33215048 10.12688/wellcomeopenres.16121.1 PMC7653641

